# Suicide. A Social Science Treatise

**Published:** 1885-09

**Authors:** 


*Suicide. A Social Science Treatise.* By W. WYNN WESTCOTT. Pp. 191. London: H. K. LEWIS. 1885.

In this small work, Dr. Wynn Westcott has presented us, in compact form, with a thoroughly scientific and highly interesting account of suicide in all its relations—historical, legal, political, and racial. Every chapter—every page, indeed—teems with interesting information, much of it bearing weighty meaning. We would particularly refer to the alarming fact that "the civilized states of Europe (with three exceptions) show a gradual and uniform increase of suicide-rate; and that, even since the beginning of the century, self-destruction has increased, and still goes on increasing, more rapidly than the increase of population, and to a greater extent than the general death-rate." One statistician has said that, "given the number of persons in the public schools of a country, he could deduce the number of suicides which took place there annually." It is a melancholy fact that increase of education increases the tendency to self-destruction.

The peculiarities of race are extraordinary. "In Europe, the highest proportion of suicide is shown by the Germanic races; and the two stocks, German and Scandinavian, divide the supremacy for the maximum rate. The *ne plus ultra* of German purity in race is found in Saxony, and this kingdom has the largest suicide ratio of any country. The height of Scandinavian purity in race is found in Denmark, and there also the ratio is very large—enormous, in fact. . . . The ratio is much smaller in the Anglo-Saxon than in the German. . . . The Celtic races avoid suicide. . . . The races of Slavonic origin show the lowest rates." These statements are supported by elaborate statistical tables.

It is even shown that races carry their peculiar suicidal propensities with them when they emigrate. In America, for instance, the excess of suicides by Germans over other races is as marked as in Europe. In respect to the influence of religion, we are told that "it is impossible to deny that the maximum rate constantly falls to Protestant states, to the Roman Catholic next, then the Greek Church, and, lastly, the minimum falls to the Jews."

Many other questions in relation to the causation of suicide are entered into and discussed with an insight and acumen worthy of all praise. Altogether, the work is one not only of profound interest, but of high scientific value.

